# Preparation and Characterization of Ultra-Fine Oil Palm Ash Powder by Ultrasonication and Alkaline Treatment for Its Evaluation as Reinforcing Filler in Natural Rubber

**DOI:** 10.3390/polym13010100

**Published:** 2020-12-29

**Authors:** Methakarn Jarnthong, Chutarat Malawet, Lusheng Liao, Puwang Li, Zheng Peng, Punyanich Intharapat

**Affiliations:** 1 Key Laboratory of Tropical Crop Products Processing of Ministry of Agriculture and Rural Affairs, Agricultural Products Processing Research Institute of Chinese Academy of Tropical Agricultural Sciences, Zhanjiang 524001, China; methakarn.jarnthong@yahoo.com (M.J.); lsliao@catas.cn (L.L.); puwangli@163.com (P.L.); pengcatas@126.com (Z.P.); 2 Faculty of Environmental Management, Prince of Songkla University, Hatyai, Songkhla 90110, Thailand; malawet.ch@gmail.com; 3 Sino-Thai International Rubber College, Prince of Songkla University, Hatyai, Songkhla 90110, Thailand; 4 Center of Excellence on Hazardous Substance Management (HSM), Bangkok 10330, Thailand

**Keywords:** oil palm ash, ultra-fine particle, ultrasonication, alkaline treatment, natural rubber composites

## Abstract

Ultra-fine oil palm ash (OPA) particles were successfully prepared using ultrasonication along with optimal chemical deagglomeration. The influence of chemical treatment by sodium hydroxide (NaOH) solution on the OPA particles was found to be an important factor in enhancing deagglomeration efficiency. The average particle size of the original OPA (41.651 μm) decreased remarkably more than 130 times (0.318 μm) with an obvious increase of Brunauer–Emmet–Teller (BET) surface area after treating the OPA with 3M NaOH, followed by ultrasonication for 30 min. The changes in particle size and surface morphology were investigated using transmission electron microscopy and scanning electron microscopy. Moreover, the chemical functional groups of the untreated and treated OPA showed different patterns of infrared spectra by the presence of sodium carbonate species owing to the effect of NaOH treatment. The incorporation of both untreated and treated OPA in natural rubber by increasing their loading can improve cure characteristics (i.e., reducing optimum cure time and increasing torques) and cure kinetic parameters (i.e., increasing the rate of cure and reducing activation energy). Nevertheless, the strength, degree of reinforcement, and thermal stability of treated OPA as well as wettability between treated OPA particles and NR were greater than that resulting from the untreated OPA.

## 1. Introduction

The utilization of oil palm ash (OPA), which is generated from the incineration of an agricultural waste from palm oil residues (e.g., palm fiber, shells, and empty fruit bunches), has gained more attention in recent years not only because it is widely available without cost, but also because of its high silica content which has specific characteristics, such as high porosity, high Brunauer–Emmet–Teller (BET) surface area, and a low coefficient of thermal expansion. These attributes result in its ability to be applied in a variety of applications such as a cement replacement material [1,2,3,4,5,6,7,8], active adsorbent material [9,10], and as a bio-filler in polymer composites [11,12,13,14,15,16,17,18,19]. 

With regard to its use as a filler function in rubber composite, it is well known that the properties of the composite material, especially the strength, are greatly controlled by the filler component. The important parameters, particle size and specific surface area, are considered to be priority factors influencing the rubber and polymer products. Therefore, although OPA is able to offer various advantages (e.g., a good source of silica, ready to use powder form, and low cost), some limitations are found, especially the large particle size corresponding to low surface area. In response to this difficulty, using OPA in a role of filler is required a trend toward smaller of particle size because of relating with the beneficial effects of increasing surface areas and dispersity to achieve mechanical properties at low filler loading [20]. 

One of the simple and practical techniques for the purpose of particles reduction is ultrasonication [21,22]. It is particularly effective in reducing the particle size and breaking up aggregates in a short treatment time and providing polydispersity of particles [23,24,25,26,27] without contaminating and destroying crystalline structure of the ash powder comparing with the ball milling method [28,29], and lower reagent is consumed comparing to the chemical methods (e.g., precipitation and sol-gel). Moreover, the advantages of uses equipment are relatively inexpensive and simple to operate and maintain. Nevertheless, reducing particle size under single ultrasonication treatment may not be able to achieve ultra-fine particle result [22]. This is because only mechanical force is applied that it is inefficient on material with various sizes and high hardness [30]. For this reason, applying developments of ultrasonication coupled with uncomplicated alkali treatments based on sodium hydroxide (NaOH) was proposed. The efficient action of the alkali treatment can induce the fragmentation of the OPA particles by attacking the hydroxide ion (OH^−^) from NaOH dissociation with siliceous phases of OPA, resulting in accessible cracking of particle. This phenomenon is therefore considered to enhance the ultrasonication efficiency by yielding ultra-fine particles as well as reducing ultrasonication treatment time and energy consumption. To our knowledge, there are no previous studies involving the use of alkali to contribute to the OPA particle size reduction combined with ultrasonication technique. 

The focus of the research was optimizing conditions to prepare ultra-fine OPA particles by applying the combination techniques of high-intensity ultrasonication and alkaline (NaOH) treatment in order to improve their characteristics and properties (e.g., particle size and shape, specific surface area, and chemical surface) with the aim of enabling the reuse of the OPA as a function of reinforcing filler in rubber composite and thereby deriving both economic and environmental benefits.

## 2. Experimental

### 2.1. Materials

**Figure 1:**
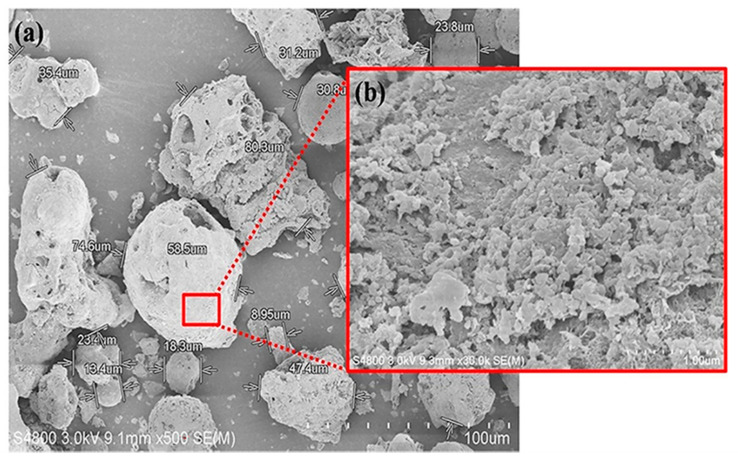
SEM micrograph of (**a**) OPA particles at 500× magnification and (**b**) surface of OPA at 30,000× magnification.

The OPA used in this research was obtained from Suksomboon Vegetable Oil Co., Ltd. in Chonburi province, Thailand. The raw OPA from the incineration of palm fiber was first ground and sieved through a 325 mesh sieve. The fine OPA powder obtained was then burnt at 550 °C in a furnace and left at room temperature, and followed by reflux in 2 wt% formic acid solution in order to remove any impurities (e.g., heavy metals). Thereafter, the chemical composition and physical properties of the OPA were determined as detailed in Table 1 and the fine OPA was found to consist of 81.55% SiO_2_. As can be seen from the scanning electron microscope (SEM) image in Figure 1, some of the OPA particles had a round shape while others were irregular with a rough and porous surface. 

Chemicals used for preparation of the unfilled and filled NR vulcanizates included zinc oxide (ZnO) and stearic acid in a role of activators, manufactured by Tianjin Shibaishi Chemical Co., Ltd., Tianjin, China and Sinopharm Chemical Reagent Co., Ltd., Beijing, China, respectively. 1,3-Diphenylguanidine (DPG) and 2,2,4-Trimethyl-1,2-dihydroquinoline (TMQ) used as a secondary accelerator and antioxidant reagent, respectively, were manufactured by Shanghai Macklin Biochemical Co., Ltd., Shanghai, China. The sulphur and N-cyclohexyl-2-benzothiazole sulfonamide (CBS) used as a vulcanizing agent and an accelerator, respectively, were manufactured by Shanghai Chemical Co., Ltd., Shanghai, China. Treated distillate aromatic extract oil (TDAE) was used as a processing aid was supported by IRPC Pub Co., Ltd., Bangkok, Thailand.

### 2.2. Ultrasonication Conditions and Chemical Treatments of OPA Particles

#### 2.2.1. The Influence of Ultrasonication Time

Samples of OPA both untreated and following chemical treatment were used in the experiments. For the chemically treated samples, prior to ultrasonication, pelleted NaOH (97%, Merck & Co., Inc., Kenilworth, NJ, USA) was used as a reagent as described below. 

For the untreated samples, 1.5 g of OPA was dispersed in 50 mL of de-ionized water and premixed for 30 min with a magnetic stirrer at a speed of 150 rpm. The suspension was placed in an ice-water bath to control the heat generated during the ultrasonication process, and the OPA was then subjected to continuous sonication treatment in an ultrasonic processor (FS-600, Shanghai Sonxi Co., Ltd., Shanghai, China) at a maximum power output of 600 W and a frequency of 20 kHz for periods of 10, 20, 30, 45, 60, 80 and 120 min. The probe tip (1.3 cm in diameter) was immersed 10-mm deep in the sample suspension.

#### 2.2.2. The Influence of Chemical Treatment by Different NaOH Solution Concentrations

The effect of the NaOH treatment on the particle size distribution of the OPA was investigated by adding 5 mL of NaOH aqueous solution with different concentrations (i.e., 1M, 2M, 3M and 4M) into the OPA suspension under stirring for 12 h at ambient temperature. The treated OPA was then ultrasonicated for 30 min in an ice-water bath at a constant power and frequency of 600 W and 20 kHz, respectively. The suspension was then centrifuged and washed with de-ionized water until the pH of leaching water was neutral, then freeze-dried for 48 h.

### 2.3. Characterization of OPA Particles

The particle size distribution after ultrasonication was determined in a range of 0.01-1000 μm by laser light scattering (Malvern Mastersizer 2000, Malvern Instruments Limited, Malvern-Worcestershire, UK). The average result was taken from at least four replications and analyzed using Mastersizer 2000 software. 

The estimation of particle size distribution in term of weight content was studied by sieve analysis with a series of sieves of square apertures of sizes 5, 10, and 20 μm. The samples were sieved and separated at least two replications.

The BET specific surface area was measured with a material characterization instrument (Model 2200A, Micromeritics Instrument Co., Norcross, GA, USA), using nitrogen as the adsorbate at liquid nitrogen temperature.

Fourier transform infrared spectroscopy (FTIR) was performed on a Nicolet FTIR spectrometer. The measurements of chemical function groups were carried out according to the KBr technique with OPA powder containing 1% in weight potassium bromide powder. The spectra were collected in a range of 4000–400 cm^−1^.

The morphology and microstructure of the OPA powder before and after ultrasonication and chemical treatment by NaOH were characterized by transmission electron microscope (TEM; JEM-1400, Hitachi, Tokyo, Japan) and a field emission scanning electron microscope (FE-SEM; S-4800, Hitachi, Tokyo, Japan).

X-ray powder diffraction (XRD) was performed using a Philips X’pert Pro Super Diffractometer (Philips, Almelo, The Netherlands) with Cu Kα radiation (λ = 1.5418 Å). The experimental conditions were fixed at 0.05° steps and 5-s accumulation time in the *2θ* range 5–90°, with testing conducted at room temperature. The average crystallite size (*D*) of the OPA particles was calculated from the XRD pattern using Scherrer Equation (Equation (1)) [31]:

*D* = *Kλ*/(*β*cos*θ*), (1)

where *D* is the average crystallite size (nm), *K* is a constant approximating to unity, *λ* is the X-ray wavelength (0.154 nm for the CuKα), *β* is the full width at half the maximum (FWHM) X-ray diffraction peak (in radians), and *θ* is the diffraction angle (in degrees).

### 2.4. Preparation of OPA Filled NR Composites

In order to investigate the reinforcement of the untreated OPA and the OPA treated with 3M NaOH in a role of filler, they were therefore mixed with NR to prepare rubber composite. The formulation of NR compounds with additives (i.e., activators, accelerator, and antioxidant), curing agent (i.e., sulphur), and fillers (i.e., untreated OPA and OPA treated with 3M NaOH) at different loading is given in Table 2. A laboratory two roll mill sized (160 × 320 mm^2^) according to ASTM designation D3184 was used for compound mixing and maintained at 70 ± 5 °C. At the end of the mixing cycle, the materials were collected and conditioned at a temperature of 25 ± 1 °C for 24 h before cure assessment. The composites of NR filled with untreated OPA and OPA treated with 3M NaOH, which their particle size distribution was determined by sieving method as detailed in Table 3. The diameter of OPA treated with 3M NaOH particles comprised mostly of 73.28 wt%, which was less than 5 μm whereas most of untreated OPA particle size distribution of 93.96 wt% occurred to equal or larger than 5 μm. This result evidently indicated that OPA treated with 3M NaOH possessed smaller particles than untreated OPA. For use in the NR compounds, they were denoted as NR/OPA-x and NR/tOPA-x, respectively, with x signifying the loading content of the filler. 

### 2.5. Characterization of OPA Filled NR Composites

Cure assessment of any individual compound such as minimum torque (M_L_), maximum torque (M_H_), torque difference (M_H_ − M_L_), scorch time (t_s2_), and optimum cure time (t_c90_) was determined, and these data also were used to calculate the cure kinetics by using a Moving die rheometer (Rheometer MDR 2000, Akron, OH, USA) at 160 °C. The amplitude of the oscillation was set at 0.5 of arc at a frequency of 1.7 Hz, according to ISO 6502. 

The mechanical properties of the NR/OPA and NR/tOPA composites in terms of their tensile strength, modulus and elongation at break were analyzed by a Hounsfield H10KS universal testing machine (Redhill, UK)based on ASTM D412 at a crosshead speed of 500 mm/min using five samples. The reinforcement index was determined by the ratio of the tensile stress at 300% strain (M_300_) to the tensile stress at 100% strain (M_100_) to indicate the reinforcing effects of the OPA on the rubber composites.

Glass transition temperature (*T_g_*) of the composites was performed using DSC Q500, TA Instrument, (New Castle, DE, USA). The sample was first heated to 100 °C with a heating rate of 10 °C min^−1^ and kept at this temperature for 5 min. This was to eliminate thermal history of the material. Then, the samples were gradually quenched to −80 °C. The second run with the same heating rate was performed, and the result was captured. 

The thermal stability of NR composites was described in term of the thermal degradation behavior. It was determined by Thermogravimetric analysis (TGA) technique using TGA Q500, TA instrument, (New Castle, DE, USA). The samples (10 mg) were placed in a platinum pan under nitrogen atmosphere. The test was then performed with a heating rate of 10 °C min^−1^ within a temperature range of 50–800 °C.

SEM (Philips XL 30-EDAX, Eindhoven, The Netherlands) was used to observe the distributions of untreated OPA and 3M NaOH-treated OPA in the composites. The fractured surfaces of NR/OPA and NR/tOPA composites were coated with gold on an ion sputter coater, and the operating voltage of the SEM was 3.0 kV with magnification 500 times and 5000 times for NR/OPA and NR/tOPA composites, respectively.

## 3. Results and Discussion

### 3.1. Effect of Ultrasonication Time

**Figure 2:**
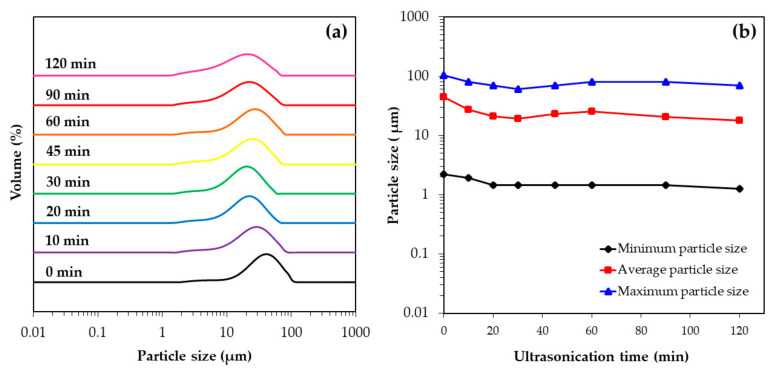
Change in particle size of untreated OPA suspension after different ultrasonication times: (**a**) particle size distribution and (**b**) the maximum particle size, average particle size and minimum particle size of untreated OPA.

The change in particle size distribution of the untreated OPA was investigated based on various ultrasonication times in a range of 10−120 min as shown in Figure 2 and summarized in Table 4. It can be seen in Figure 2a that the average particle size of the untreated OPA was reduced from 41 μm to 26 μm after ultrasonication for 10 min, and further reduced to 19 μm after 30 min of ultrasonication. However, with increases in the ultrasonication time beyond 30 min the average particle size tended to slightly increase, or remain almost unchanged even if ultrasonication was continued for up to 120 min. The particle size distribution showed that, after ultrasonication, most of the untreated OPA particles ranged in size between 18 and 26 μm (Table 4). The maximum, average, and minimum particle size of the OPA with different ultrasonication times are shown in Figure 2b. The results showed that the particle size of the untreated OPA was in a range of 1−100 μm, with no particles smaller than 1 μm being detected. Therefore, employing ultrasonication can reduce the particle size of untreated OPA down to approximately 50% of its original size at the optimum ultrasonication time of 30 min. Prolongation of ultrasonication beyond this time did not further decrease the size of the OPA particles.

### 3.2. Effect of Chemical Treatment by NaOH

**Figure 3:**
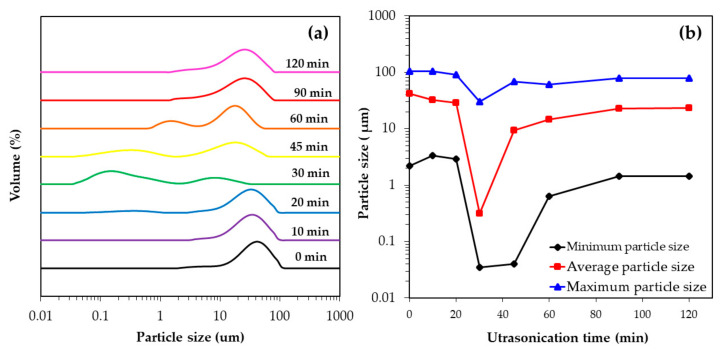
Change in particle size of the 3M NaOH treated OPA suspension before different ultrasonication times: (**a**) particle size distribution and (**b**) the maximum particle size, average particle size and minimum particle size of treated OPA.

In order to improve the deagglomeration efficiency of untreated OPA particles, the OPA was treated with 3M NaOH solution for 12 h before ultrasonication at different times. As can be seen in Figure 3a, the particle size distribution of the treated OPA was bi-modal with ultrasonication times of 30 min and beyond, with the presence of large agglomerates (>5 μm) and smaller fragments (<1 μm) especially at 30 and 45 min of ultrasonication. The minimum particle size of the treated OPA was observed at 35 nm (Figure 3b). However, a further 30 min ultrasonication caused the average particle size to increase as shown in Table 4. The increase in the number of large particles and the decrease in small particles was due to the reagglomeration of the OPA particles to a level similar to the initial size distribution.

From the results, it can be clearly seen that the degree of deagglomeration of OPA after it was treated with NaOH, followed by 30 min of ultrasonication was greater than that of untreated OPA, which can be explained by the progressive reduction in the particle size of treated OPA being enhanced by the breakage of Si-O-Si bonds after pretreatment with NaOH [32] associated with ultrasonic cavitation effects, which caused the agglomerated particles to fragment into smaller particles. However, longer ultrasonication times allowed the reagglomeration of some broken NaOH-treated OPA particles into groups and the formation of large agglomerates [24]. It is worth noting that particle adhesion increases when the particle size decreases and similar results have been reported in earlier studies [25,26], with an initial rapid size reduction being followed by lower size reductions or even reagglomeration of the particles with continued ultrasonication.

**Figure 4:**
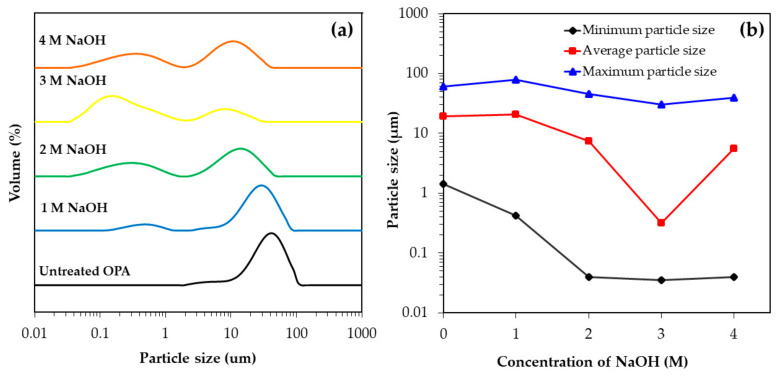
Change in particle size of OPA treated with NaOH at different concentrations followed by ultrasonication for 30 min: (**a**) particle size distribution and (**b**) the maximum particle size, average particle size and minimum particle size of treated OPA.

The effect of different NaOH concentrations of 1M, 2M, 3M and 4M on the particle size distribution of OPA following ultrasonication was investigated and the results are shown in Figure 4 and summarized in Table 5. It can be seen that the particle size of OPA treated with NaOH at all concentrations showed an obviously bi-modal distribution pattern with one peak for particle sizes larger than 5 μm and another for particle sizes smaller than 1 μm. On the other hand, the OPA without treatment by NaOH had an absolutely unimodal distribution, with a peak for particles larger than 10 µm. In the case of the treated OPA, the population of small agglomerates with a particle size less than 1 μm increased with increasing NaOH concentrations from 1M to 3M, which clearly indicated the progression of the deagglomeration of the OPA particles from large to small. The smallest OPA particle size was found under the condition of 3M NaOH treatment at an ultrasonication time of 30 min, and was in a range of 0.035–30.200 μm, with an average particle size of 0.318 μm (Table 5). Nevertheless, the average particle size increased when the OPA was treated with 4M NaOH. This can be explained by the reagglomeration of the OPA particles at a high concentration of NaOH due to the strong hydrogen bonds of the hydroxyl groups on the surface of the freshly broken OPA particles. In addition, the reagglomeration of the OPA affects the value of the BET surface area as shown in Table 5. It was found that the surface area of the OPA after treatment by 3M NaOH with ultrasonication for 30 min increased more than three times from 45.6 m^2^/g to 150.3 m^2^/g. Nevertheless, when the concentration of NaOH increased from 3M to 4M, the surface area of the treated OPA particles decreased to 115.2 m^2^/g. This result undoubtedly corresponds to the increase in particle size due to the reagglomeration previously mentioned, leading to a decrease in the surface area of the OPA particles.

**Figure 5:**
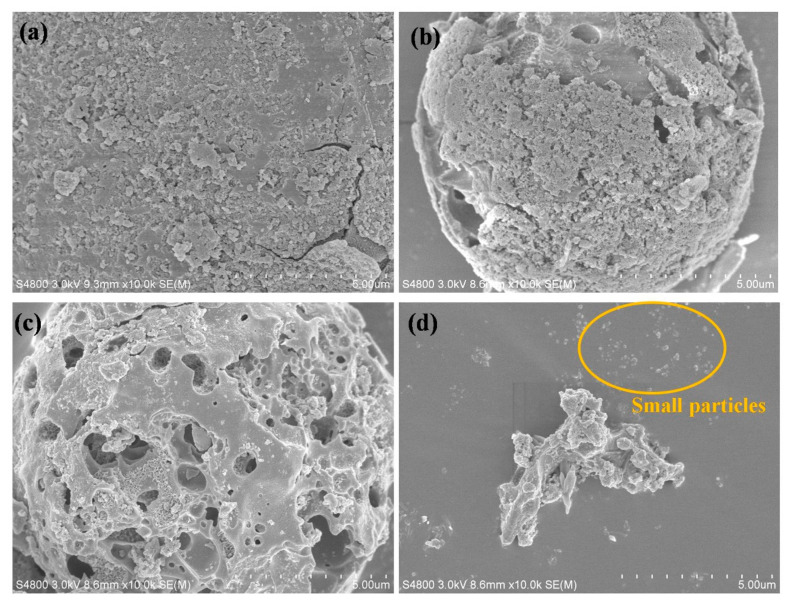
FE-SEM images of OPA particles at different concentrations of NaOH treatment after 30 min ultrasonication: (**a**) untreated OPA, (**b**) 1M NaOH treated OPA, (**c**) 2M NaOH treated OPA and (**d**) 3M NaOH treated OPA.

The effect of NaOH concentrations of 1M, 2M and 3M on the morphology of OPA particles was investigated by SEM as shown in Figure 5. It can be observed that after treatment with 1M NaOH (Figure 5b), some part of OPA surface morphology became smoother with larger pore than that of the untreated OPA (Figure 5a). However, after treatment with 2M NaOH (Figure 5c) the surface morphology was quite different from that resulting from treatment with 1M NaOH (Figure 5b). The outer surface of the 2M NaOH-treated OPA displayed more prominent of spongy surface than the surface of the 1M NaOH-treated OPA. In the case of the 3M NaOH-treated OPA (Figure 5d), the OPA particle markedly broken up into small particles, and some portion of irregular shape also occurred. These changes in the morphology of the OPA particles (i.e., changes in the size and surface appearance) could be due to the alkali action. 

In this study, it was found that the pretreatment of OPA agglomerates by NaOH induced fragmentation of the OPA particles, and enhanced the deagglomeration when ultrasonication was applied because with the presence of NaOH solution. The surface ☰Si-OH groups on the OPA were ionized according to the following reaction [32]:

$$☰\mathrm{Si-OH}+\mathrm{NaOH}\;\to \;☰{\mathrm{Si-O}}^{-}{\mathrm{Na}}^{+}+H_{2}O$$

At low concentrations of NaOH (1M and 2M NaOH), only the outer surface was negatively charged due to ionized ☰Si-O^−^ groups. This caused the erosion and silicate dissolution at the surface of the OPA particles, resulting in more larger pore structure appearance on the surface of the 1M and 2M NaOH-treated OPA, as can be seen in Figure 5b,c respectively. However, at higher concentrations of NaOH solution, the ionic strength and concentration increased leading to deeper penetration by OH^−^ ions into the agglomerates which ionized the inner ☰Si-O-Si☰ bonds [32,33] resulting in a product which could be further hydrolyzed to form a silanol group on the particle surface as described below:

$$☰\mathrm{Si-O-Si}☰+2\mathrm{NaOH}\;\to \;2☰{\mathrm{Si-O}}^{-}{\mathrm{Na}}^{+}+2H_{2}O\;☰{\mathrm{Si-O}}^{-}{\mathrm{Na}}^{+}+H_{2}O\;\to \;\mathrm{Si-OH}+\mathrm{NaOH}$$

**Figure 6:**
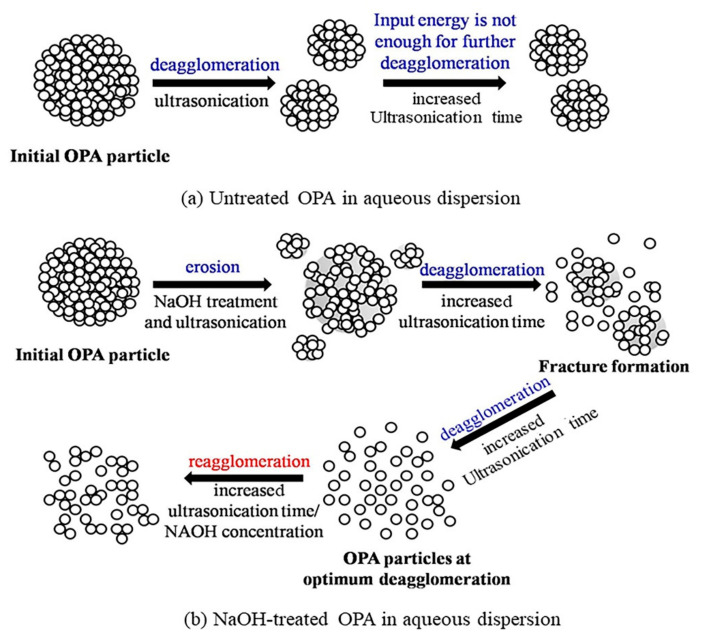
Schematic diagram showing the changes in particle size and morphology of (**a**) untreated OPA under ultrasonication and (**b**) NaOH-treated OPA, as a function of ultrasonication time.

This reaction enhanced the fracturing process during ultrasonication due to the propagation of cracks from defects inside the particles, which accelerated the deagglomeration of the OPA particles into smaller particles and increased the number of small-sized particles (<1 μm) in the system. However, silanol group formation from the hydrolysis of ☰Si-O^−^Na^+^ can cause interaction among the particles or their agglomerates leading to reagglomeration. A schematic representation of the change in particle size of the OPA during ultrasonication is shown in Figure 6.

**Figure 7:**
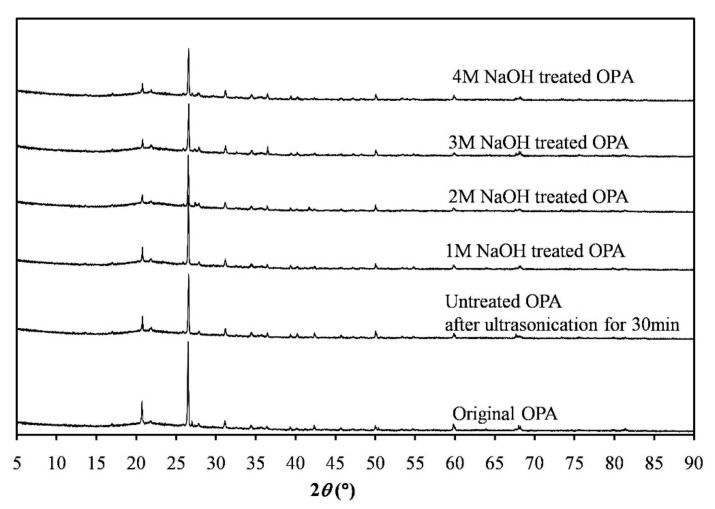
XRD patterns of original OPA, untreated OPA by ultrasonication for 30 min and treated OPA with NaOH at different concentrations followed by ultrasonication for 30 min.

The XRD patterns of raw OPA, untreated OPA after ultrasonication for 30 min and OPA treated with NaOH at different concentrations with ultrasonication for 30 min were derived in order to examine their crystalline structure as shown in Figure 7. Clear quartz peaks appeared in all the diffractograms and those peaks were narrow and intense, indicating that this OPA was well-crystallized. Major diffraction peaks at 2*θ* were detected at angles of 20.8° (010), 26.6° (011), and 50.0° (012), which corresponded to α-quartz (SiO_2_), and accorded well with the chemical composition of the OPA (Table 1). In addition, the XRD pattern of the OPA powders after chemical treatment with NaOH and/or ultrasonication resembled the pattern of the original material, which implied an unchanged structure. This result is in agreement with a previous study [34], which reported that the crystalline structure of OPA remained unchanged after heat treatment and grinding. The average crystallite size is also shown in Table 5. It can be seen that the crystallite size of the untreated OPA slightly decreased after ultrasonication for 30 min and noticeably decreased in the NaOH-treated OPA after ultrasonication. The smallest average crystal size of the treated OPA was obtained using 3M NaOH treatment with ultrasonication, which reflected the diminishing degree of structural perfection of the material after NaOH treatment.

**Figure 8:**
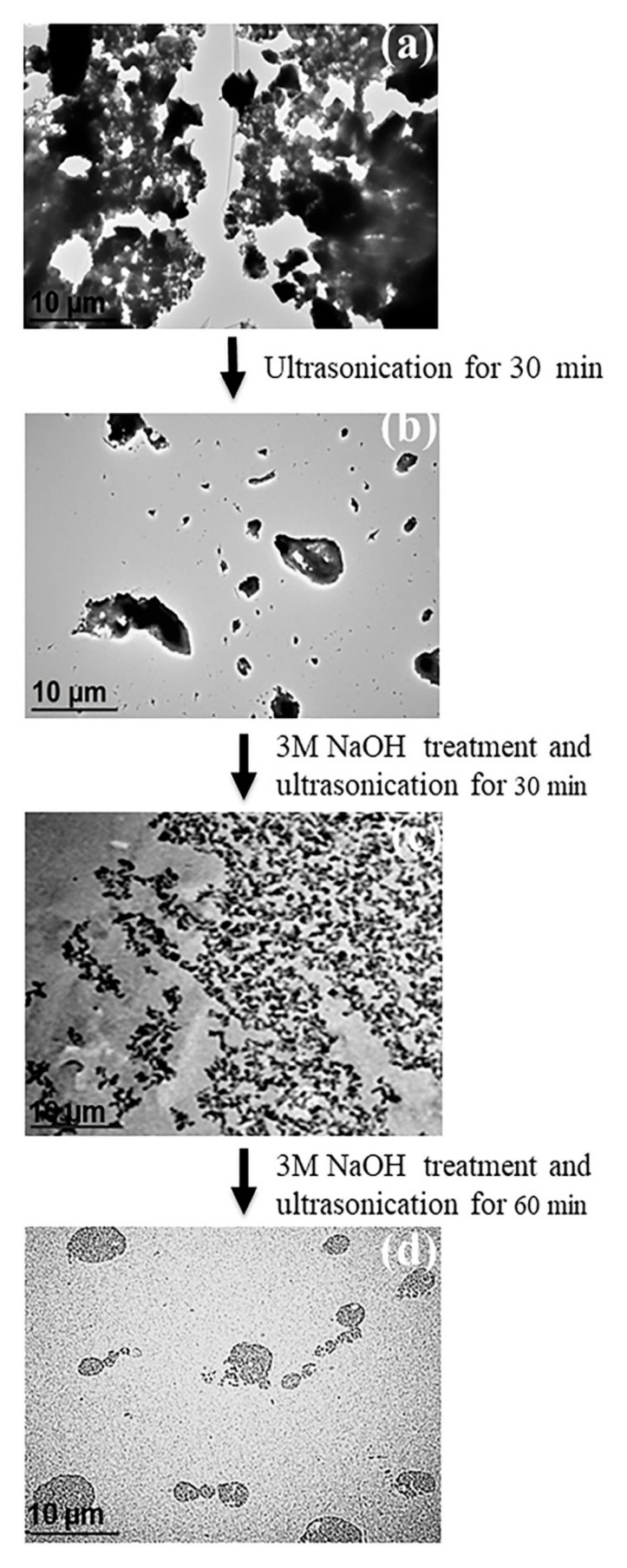
TEM micrographs of OPA particles: (**a**) original OPA, (**b**) untreated OPA after ultrasonication for 30 min, (**c**) 3M NaOH-treated OPA followed by 30 min ultrasonication and (**d**) 3M NaOH-treated OPA followed by 60 min ultrasonication.

The deagglomeration and reagglomeration of the treated OPA particles during ultrasonication was confirmed by the TEM micrographs as shown in Figure 8. It can be seen that the original OPA without ultrasonication (Figure 8a) contained large particles and formed large agglomerates whereas, ultrasonication for 30 min of the untreated OPA caused the agglomerates to break and split into smaller agglomerates (Figure 8b). However, the deagglomeration during the ultrasonication of the OPA particles was more effective when it was accompanied by treatment with 3M NaOH (Figure 8c), which indicated that the siloxane bonds between the particle surfaces were not broken easily by ultrasonication without chemical treatment. Nevertheless, the reagglomeration of the broken treated OPA particles was clearly seen to increase the particle size when the ultrasonication was prolonged to 60 min (Figure 8d). This may be attributed to the strong interaction of the silanol groups among the particles. 

**Figure 9:**
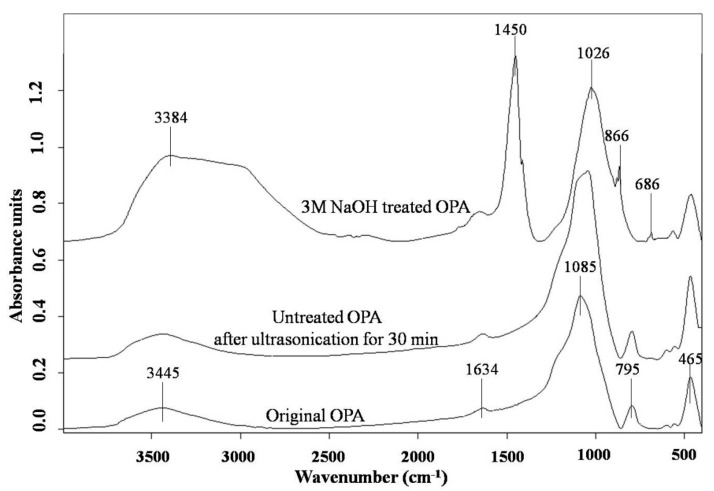
FTIR spectra of original OPA, untreated OPA after ultrasonication for 30 min and 3M NaOH-treated OPA followed by ultrasonication for 30 min.

FTIR spectra of untreated OPA and NaOH treated OPA were obtained and compared to that of original OPA in order to determine changes in the chemical function groups of the materials. The characteristic peaks of them are shown in Figure 9 and described in Table 6.

As can be observed in Figure 9, the FTIR spectra of original OPA and untreated OPA after ultrasonication showed similar patterns with infrared absorption bands at 465, 795, 1085, 1634, and 3445 cm^−1^, corresponding to the vibration mode assignment for silica. This agreed with the results of the chemical composition analysis as well as those of the XRF and XRD analyses, that the OPA in this study consisted mostly of SiO_2_. The broad band observed in the region of 3100–3500 cm^−1^ and the peak at 1634 cm^−1^ are the symmetric and asymmetric stretching of the O-H vibration of the silanol (Si-OH) in the OPA. It is also notable that, after treatment with NaOH, the peak intensity in the range of 3100–3500 cm^−1^ and 1634 cm^−1^ (the deformation mode of H-O-H) greatly increased due to the increase in the silanol groups in the treated OPA particles resulting from the increased surface area. In addition, the shift of the Si-O-Si vibration of untreated OPA at wavenumber 1085 cm^−1^ to a lower wavenumber (1026 cm^−1^) after treatment with NaOH suggested that oxygen had connected to Na^+^ ions to produce sodium silicate (☰Si-O^−^Na^+^) [38] with the appearance of Si-O bending vibration of the Na–O occurring at 686 cm^−1^. In addition, it is interesting to note that the FTIR spectrum of the NaOH-treated OPA contained a strong carbonate band at 1450 cm^−1^ in the asymmetric stretch, with an absorption peak at 866 cm^−1^ in the out-of-plane bend. These peaks are characteristic of sodium carbonate (Na_2_CO_3_) species, which is a product of the carbonation of sodium silicate (Na_2_SiO_3_) and carbon dioxide (CO_2_) in the atmosphere according to the following reaction [35,39]:

$${\mathrm{Na}}_{2}{\mathrm{SiO}}_{3}+{\mathrm{CO}}_{2}\;\to \;{\mathrm{Na}}_{2}{\mathrm{CO}}_{3}+{\mathrm{SiO}}_{2}$$

### 3.3. Properties of OPA Filled NR Composites

#### 3.3.1. Cure Characteristic and Cure Kinetics

**Figure 10:**
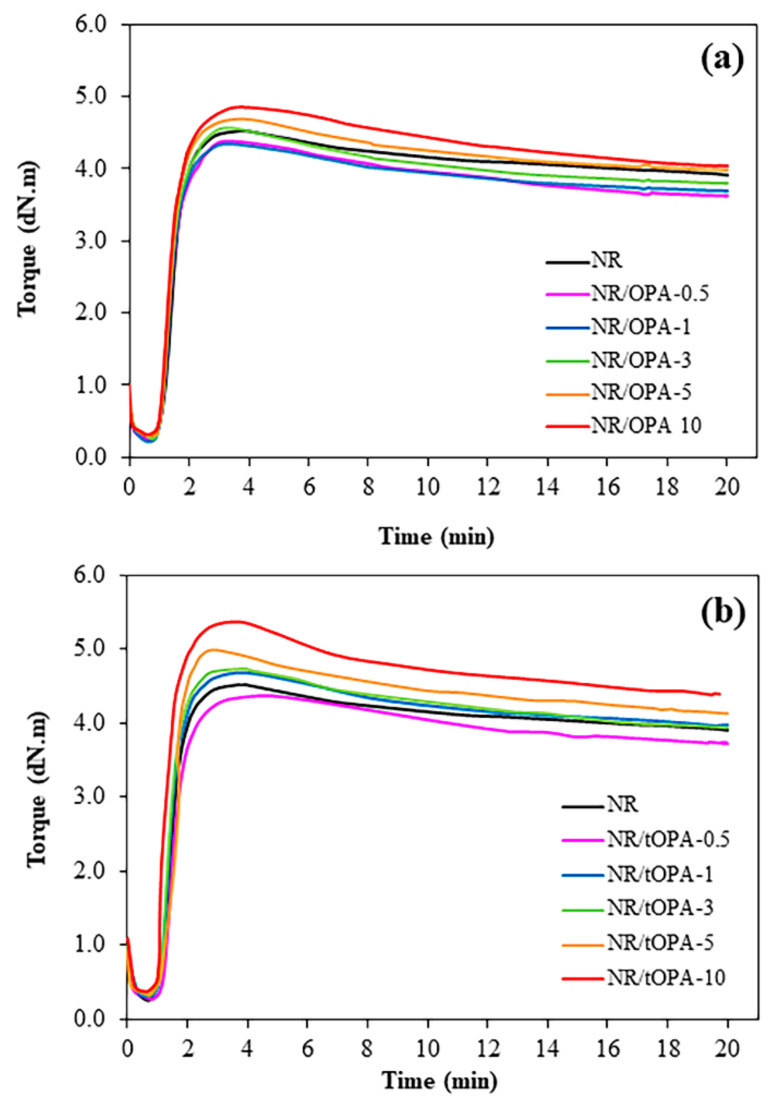
Cure curves of (**a**) NR/OPA and (**b**) NR/tOPA at various filler loadings.

Figure 10a,b shows cure curve behavior of NR/OPA and NR/tOPA compounds, respectively, and the cure data are described in Table 7 in terms of the minimum torque (M_L_), maximum torque (M_H_), torque difference (M_H_ − M_L_), optimum cure time (t_c90_), scorch time (t_s2_), and cure rate index (CRI). It can be seen that NR filled with both untreated OPA and 3M NaOH-treated OPA tended to increase the torque values (M_L_, M_H_, and M_H_ − M_L_) with increasing their loading. The increase of M_L_ of the filled compounds indicated that the incorporation of both fillers into the rubber matrix led to the higher viscosity to the rubber composites due to the presence of their causing the increase of resistance to the flow of rubber molecules. For t_c90_ and t_s2_ of the filled compounds, they provided shorter with higher CRI than the unfilled one, and markedly reduced t_c90_ and t_s2_ at high loading, which was similar to previous studies [40]. This behavior resulted from the base effect of oil palm ash, which accelerated the vulcanization process. Therefore, CRI, which is directly related to the speed of the vulcanization reaction also increased. Considering the different fillers between untreated OPA and 3M NaOH-treated OPA filled NR, it was found that NR/tOPA showed obviously trend in higher M_L_, MH, and M_H_ − M_L_ and shorter t_c90_ and t_s2_ with higher CRI than NR/OPA of whatever the filler loading. It should be interesting to note that the presence of strong base Na_2_CO_3_ observed only for tOPA, which is from the effect of chemical treatment performing as evident from FT-IR result can also contribute to vulcanization process [41]. The participation of this component led to a considerably greater curing rate and crosslink density relative to the increase of M_H_ and M_H_ − M_L_ than untreated OPA filled NR.

For cure kinetic of rubber, it was focused only on determination of vulcanization step. Since, the early stage of this region is linear. In addition, the rate of cure and activation energy of vulcanization reaction are able to determine by using first order kinetic [42]. From the cure data, the rate of cure (α) can be calculated using the following Equation (2):

(2) $$\alpha =\frac{M_{t}-M_{min}}{M_{max}-M_{min}}$$

where *M_t_*, *M_min_*, and *M_max_* are the torque at the time t, the minimum torque, and the maximum torque, respectively.

The crosslink kinetic parameters were determined by using the first order reaction at the temperature of 160 °C as shown in this Equation (3):

(3) $$\ln\left[1-\alpha \right]=-\mathrm{kt}+\ln k_{0}$$

where 1 − α is the conversion ratio, k is cure rate constant, k_0_ is the frequency factor, and t is time. From this equation, k and lnk_0_ can be obtained from slope and intercept of the graph, respectively.

The activation energy of vulcanization reaction in the curing stage can be evaluated through the Arrhenius Equation (4):

(4) $$k=k_{0}\exp\;\left(-\frac{E_{a}}{\mathrm{RT}}\right)$$

where k is the rate of reaction, k_0_ is the frequency factor, E_a_ is the activation energy, R is the universal gas constant, and T is the absolute temperature.

**Figure 11:**
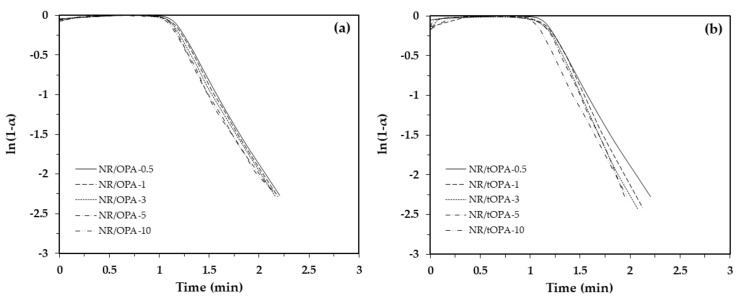
Plot ln[1 − α] versus time of (**a**) NR/OPA and (**b**) NR/tOPA at different loading in vulcanizing stage.

From Figure 11, it can be seen that the plot of the conversion ratio with time shows the linear regions corresponding to first order kinetic. Therefore, the cure kinetic of NR/OPA and NR/tOPA was able to determine rate of cure (k) and activation energy (E_a_) of vulcanization reaction by using Arrhenius equation following Equation (4). The cure kinetics parameters and activation energy of OPA and tOPA filled natural rubber, which were determined at temperature of 160 °C, are summarized in Table 8.

The rate of vulcanization and activation energy of NR/OPA and NR/tOPA at various loading were described in Table 8. It was found that NR/tOPA showed the trend of higher rate of vulcanization reaction and lower activation energy than NR/OPA at a given loading. This indicated that the vulcanization reaction of tOPA filled NR was able to be stimulated due to the alkaline nature by the presence of strong base Na_2_CO_3_ of tOPA as mention previously, which was able to accelerate vulcanization resulting in the reduction in activation energy for initiating the reaction. In addition, the presence of high filler loading of both OPA and tOPA therefore provided a decrease trend of activation energy due to the acceleration effect of NR with the increasing fillers loading.

#### 3.3.2. Tensile Properties

**Figure 12:**
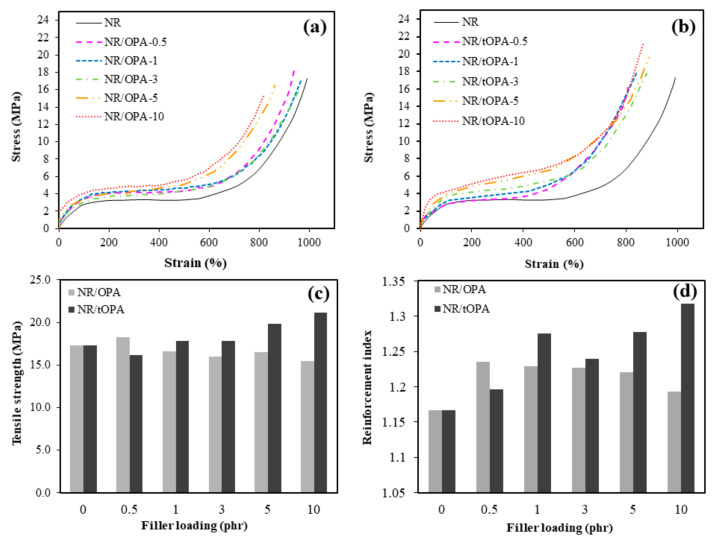
Stress-strain curves of (**a**) NR/OPA and (**b**) NR/tOPA; (**c**) tensile strength and (**d**) reinforcement index of NR/OPA and NR/tOPA at various filler loadings.

The effect of filler loading (i.e., 0.5, 1, 3, 5 and 10 phr) and chemical treatment of OPA on the mechanical properties in the presence of typical stress-strain curves of NR composites is shown in Figure 12. It can be seen, comparing between filled and unfilled rubber, that the filled rubber composites with both untreated OPA and 3M NaOH-treated OPA at all loadings showed obviously higher modulus (the initial slope of the stress-strain curve) and tensile strength (ultimate tensile stress) than the unfilled rubber, while the elongation at break (strain at break) of most rubber composites tended to decrease with increasing quantities of filler. This is well known to be due to the filler causing increased stiffness and decreased elasticity of rubber matrix. Regarding between the NR filled with untreated and treated OPA, it can be observed that the composites with untreated OPA showed the highest tensile strength at the lowest loading (0.5 phr; see Figure 12c) [11,43] but the tensile strength decreased thereafter when the OPA loading was further increased. A similar result was found in OPA-filled vulcanized NR as reported by Ooi et al. [43]. Meanwhile, the tensile strength of the NR filled with tOPA increased with increasing filler loading, reaching a maximum with the highest tOPA loading used (10 phr) as can be seen in Figure 12c. This result indicated that OPA treated with 3M NaOH and then ultrasonicated can enhance the polymer-filler interaction corresponding to a higher reinforcement index as compared to that of the NR/OPA composites as shown in Figure 12d. It suggested that the smaller particle size with a larger surface area and a rough abrading surface, which was noted for the treated OPA, promoted wettability at the interface and interactions by physical adsorption and penetration of the rubber molecules at the exterior OPA surface. These actions resulted in a better polymer–filler interaction, leading to an increase in the level of the reinforcement index.

#### 3.3.3. Glass Transition Temperature

**Figure 13:**
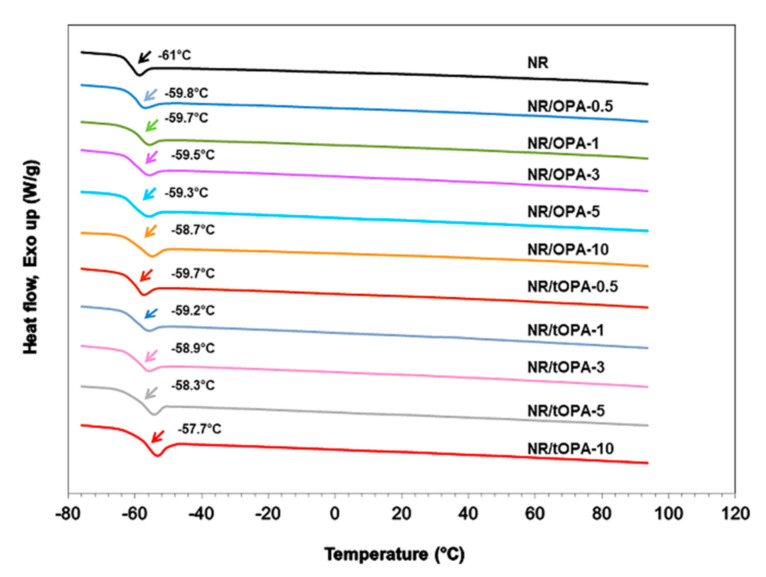
DSC thermogram of unfilled NR and NR/OPA and NR/tOPA at various filler loadings.

In order to evaluate the effect of modify physical properties of OPA after treatment with NaOH accompanying with ultrasonication compared with untreated OPA, which was only applied by ultrasonication, in rubber composite, glass transition temperature (*T_g_*) is a well-known parameter to measure this effect by relating to the temperature at initiating rubber chain mobility. Figure 13 shows *T_g_* of NR, NR/OPA, and NR/tOPA vulcanizates recorded by DSC thermogram. It can be seen that *T_g_* of unfilled NR appeared at the lowest temperature of −61 °C. Meanwhile, the filled NR (NR/OPA and NR/tOPA) was observed their *T_g_* at higher temperature in a range of −59.8 °C to −57.7 °C, and it increased with increasing their loading. The shift in *T_g_* toward at higher temperature when OPA and tOPA, whatever their loading, was incorporated in NR matrix. It is attributed to increasing the viscosity and stiffness of the rubber composite resulting the decrease of flexibility of part of rubber chains. Considering the different type of fillers, it can be observed that a greater incremental increase of *T_g_* was noted for the introduced 3M NaOH-treated OPA into the rubber matrix than untreated OPA. This result indicated that the filled NR with 3M NaOH-treated OPA provided better interaction at the interface between rigid particle and rubber matrix due to possessing a higher specific surface area and participation of inorganic compounds, especially strong alkali base of Na_2_CO_3_ in the vulcanization reaction to contribute crosslink density. These behaviors caused limited rubber chain flexibility and mobility, leading to the increase of *T_g_*. Therefore, this evidence can indicate the advantage of applying ultrasonication coupled with alkali treatments for modifying OPA physicochemical properties.

#### 3.3.4. Thermal Stability

**Figure 14:**
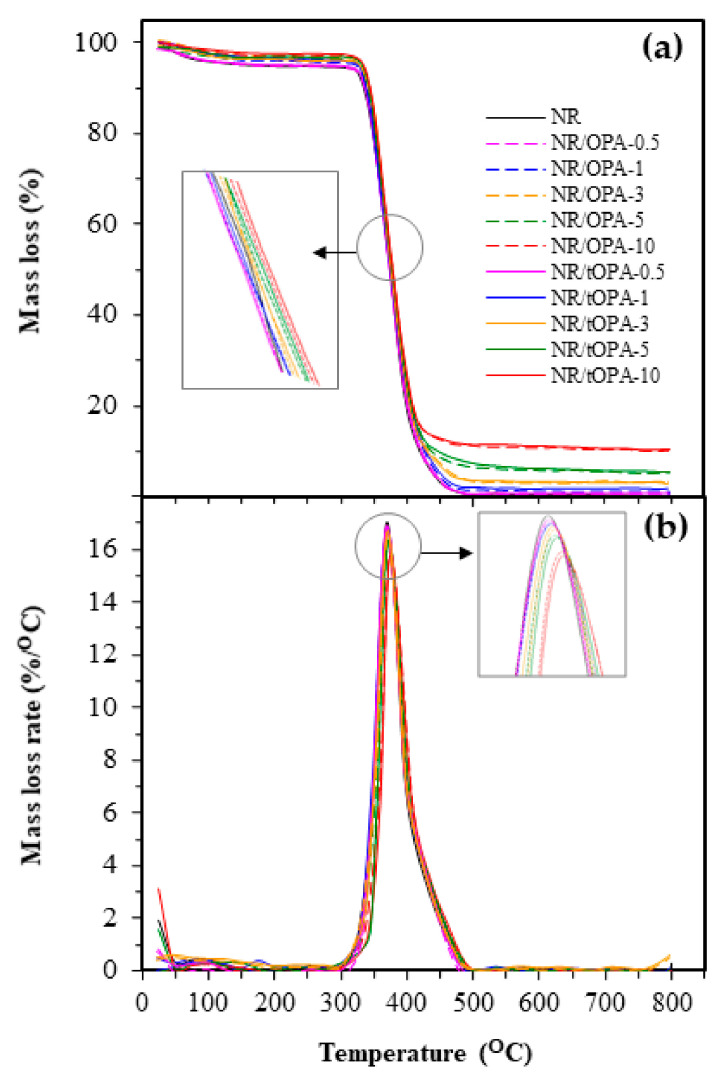
(**a**) TGA and (**b**) DTG curves of unfilled NR and NR/OPA and NR/tOPA at various filler loadings.

Thermal analysis was used to determine thermal stability under nitrogen atmosphere of particular NR composites occurring under an imposed change in temperature. TGA and DTG curves of the unfilled NR and NR composites (i.e., NR/OPA and NR/tOPA) with different filler loading at 0.5, 1, 3, 5, and 10 phr were shown in Figure 14a,b, respectively. It can be seen that the weight loss in the degradation pattern of them showed one main step decomposition corresponding to the degradation of NR matrix with a range of total weight loss of 99.7–89.5% depending on the filler loading in rubber composites. The initial decomposition temperature (*T*_onset_) of the composites were summarized in Table 9. It can be seen that *T*_onset_ of untreated OPA and 3M NaOH-treated OPA filled NR of whatever loading slightly shifted towards to be higher temperature in a range of 312.7–335.5 °C than NR without filler corresponding to *T*_onset_ of 310.3 °C, and *T*_onset_ of both fillers filled NR increased with increasing their loading. It designated that NR composites containing OPA and tOPA displayed the improvement of thermal stability. This behavior can be explained by the fact that the low starting decomposition temperature of fillers was over than NR matrix. In addition, the presence of OPA and tOPA in rubber can act as a barrier. These conducts therefore can retard the thermal penetration as well as heat distribution in the matrix. Comparing between NR/OPA and NR/tOPA, *T*_onset_ and the temperature at the maximum rate of weight loss (*T_max_*) which was decided according to the peak of DTG thermogram (Figure 14b) showed that NR/tOPA provided slightly higher *T*_onset_ and *T*_max_ than NR/OPA at a given loading. From the result, it could be ascribed to the effect of the incorporation of tOPA into the matrix of NR, which promoted the interfacial area as discussed earlier. This characteristic caused increasing interaction and strengthens interfacial adhesion between dispersed tOPA particles and NR matrix. Therefore, more energy was required to destroy the filler–matrix interaction, resulting in more thermal stability of the composites. Similar observations were also reported [44,45,46,47].

#### 3.3.5. Morphological Properties

**Figure 15:**
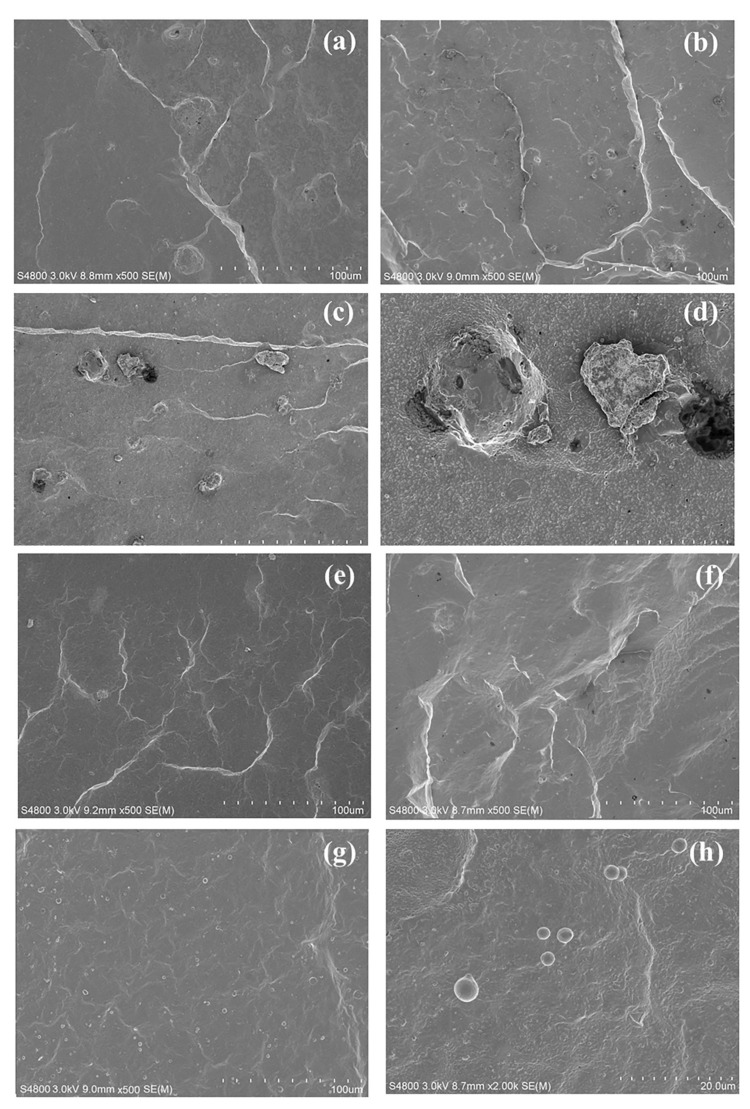
FE-SEM images of NR/OPA at different loading levels of (**a**) 1 phr, (**b**) 5 phr and (**c**) 10 phr at 500× magnification and (**d**) 10 phr at 2000× magnification and NR/tOPA at different loading levels of (**e**) 1 phr, (**f**) 5 phr and (**g**) 10 phr at 500× magnification and (**h**) 10 phr at 2000× magnification.

The FE-SEM micrographs of the fractured surface of NR/OPA and NR/tOPA at loading levels of 1, 5, and 10 phr are shown in Figure 15a–f. It can be seen that the untreated OPA (Figure 15a–c) obviously revealed larger particle size than treated OPA (Figure 15e–g) in the NR matrix. In addition, some dispersed untreated OPA particles appeared with voids apparent at the interface between them and rubber matrix (Figure 15d), indicating poor interfacial adhesion. The presence of these voids could act as points of weakness in the rubber composite which would tend to initiate stress concentration during applied stress, leading to a decrease in the strength of the rubber composite. Meanwhile, in the case of the NR/tOPA composite (Figure 15e–g), smaller particle size, better dispersion, and embedment of tOPA in the NR matrix can be observed. This was due to the chemical surface and small size of the tOPA particle after chemical treatment, which enhanced wettability between dispersed OPA particles and NR matrix as shown in Figure 15h. This leads to improve stress transfer between the rubber-filler phases and provides greater strength, which is in agreement with the findings in this study in relation to the tensile properties and reinforcement index (Figure 12).

## 4. Conclusions

An effective approach was successfully developed for producing ultra-fine OPA with the smallest particle and an approximate average particle size of 35 nm and 300 nm, respectively. The most effective deagglomeration of OPA was obtained by chemical treatment with 3M NaOH followed by ultrasonication for 30 min. Nevertheless, the polydispersity of resulting particles also occurred and optimal protocols should therefore be followed to achieve a narrower distribution of the particles. For the morphology of untreated OPA particles, after ultrasonication, it was shown that they were spherical in shape with a rough surface. After treatment with NaOH, both spherical and irregular shaped particles of OPA were found with a porous surface, indicating both erosion and fracture mechanisms during ultrasonication. The FTIR and XRD results revealed an enrichment of the SiO_2_ content in the OPA, and the ultrasonication did not cause any changes in its chemical or crystalline structure. Regarding the reuse of the untreated OPA and 3M NaOH-treated OPA as the function of filler in rubber composite, cure characteristics of NR/tOPA exhibited different from NR/OPA by providing shorter scorch time and cure time with greater curing rate and torques at whatever loading. The cure kinetic of both rubber compounds corresponded to first order kinetic, and the rate of cure and activation energy of vulcanization reaction of NR/tOPA showed the trend of higher value and lower activation energy, respectively, than another one at whatever loading due to the alkaline character by the presence of strong base Na_2_CO_3_ of tOPA. The tensile properties and thermal stability of NR/tOPA were also superior to these properties of whatever loading over OPA/NR and unfilled NR, respectively. In addition, a greater incremental increase of *T_g_* was noted for the introduced 3M NaOH-treated OPA into the rubber matrix than untreated OPA. The morphology of dispersed 3M NaOH-treated OPA particles in NR matrix showed an obviously smaller particle size with better dispersion, good embedment, and greater wettability at the interface than the untreated one.

## Figures and Tables

**Table 1:** Chemical composition of fine OPA powder.

Component	Weight (%)
MgO	1.69
Al_2_O_3_	2.12
SiO_2_	81.55
P_2_O_5_	4.93
K_2_O	2.72
CaO	3.21
TiO_2_	0.16
MnO	0.09
Fe_2_O_3_	2.90
CuO	0.04
SrO	0.02
ZrO_2_	0.03
Density (g/cm^3^)	2.24
Specific surface area (m^2^/g)	45.60

**Table 2:** The compound formulation and mixing schedule of unfilled and filled NR with untreated OPA (OPA) and 3M NaOH-treated OPA (tOPA) at different loading.

Ingredients	Quantity (phr)	Mixing Step	Mixing Time (min)
NR	100	Add NR for Mastication	0:00
ZnO	5	Add ZnO and stearic acid	5:00
Stearic acid	1		
OPA or tOPA	0.5, 1, 3, 5 and 10	Add filler (OPA or tOPA) and TDAE oil	8:00
TDAE oil	8		
TMQ	1	Add TMQ	15:00
DPG	1	Add DPG	16:00
CBS	1.5	Add CBS	17:00
Sulfur	1.5	Add sulfur	18:00
		dumped and left at room temperature	20:00

**Table 3:** Particle size distribution of untreated OPA and OPA treated with 3M NaOH.

Diameter (μm)	Untreated OPA		OPA Treated with 3M NaOH	
	Particle Size Distribution (wt%)	Accumulated Weight (%)	Particle Size Distribution (wt%)	Accumulated Weight (%)
>20	64.30	64.30	1.72	1.72
20−10	14.70	79.00	3.79	5.51
10–5	14.96	93.96	21.21	26.72
<5	6.04	100	73.28	100

**Table 4:** Average particle size of OPA before and after chemical treatment.

Ultrasonication Time (min)	Average Particle Size (μm)	
	Untreated OPA	3M NaOH Treated OPA
0	41.651	41.651
10	26.971	32.697
20	21.187	28.753
30	19.358	0.318
45	22.872	9.470
60	25.603	14.464
90	20.570	22.664
120	18.050	23.283

**Table 5:** Average particle size, range of particle size and BET surface area of untreated OPA and treated OPA with NaOH at different concentrations followed by ultrasonication for 30 min.

OPA Samples	Ultrasonication Time (min)	Range of Particle Size (μm)	Average Particle Size (μm)	BET Surface Area (m^2^/g)	Average Crystallite Size (nm)
Original OPA	0	2.188−104.713	41.651	45.6	54.17
Untreated OPA	30	1.445–60.256	19.358	76.0	52.30
1M NaOH treated OPA	30	0.423–79.433	20.915	84.0	45.10
2M NaOH treated OPA	30	0.041–45.709	7.403	119.4	39.45
3M NaOH treated OPA	30	0.035–30.200	0.318	150.3	32.73
4M NaOH treated OPA	30	0.040–39.811	5.619	115.2	47.93

**Table 6:** FTIR frequencies of both untreated OPA and 3M NaOH-treated OPA samples.

Vibration Frequencies (cm^−1^)	Assignment	References
3500–3100 and 1634	O-H stretching vibration	[19,35,36]
1450 and 866	Na_2_CO_3_	[36,37]
1100−1000	Si-O-Si stretching vibration	[31,35,36]
795	Si-O-Si stretching vibration or Si-O-Al	[19,31,35,36]
686	${\mathrm{CO}}_{3}^{2-}$ from Na_2_CO_3_	[36]
465	Si-O-Si bending vibration	[31,35,36]

**Table 7:** Curing characteristics of unfilled NR and OPA and tOPA filled NR compounds with different loading.

Sample	M_L_ (dN.m)	M_H_ (dN.m)	M_H_ − M_L_ (dN.m)	Cure Time, t_c90_ (min)	Scorch Time, t_s2_ (min)	CRI (1/min)
NR	0.26	4.52	4.26	2.24	1.14	90.91
NR/OPA-0.5	0.26	4.37	4.11	2.25	1.10	86.95
NR/OPA−1	0.28	4.34	4.06	2.22	1.08	87.72
NR/OPA-3	0.30	4.57	4.27	2.14	1.06	92.59
NR/OPA-5	0.32	4.67	4.35	2.00	1.06	106.38
NR/OPA−10	0.33	4.89	4.56	1.85	1.02	120.48
NR/tOPA-0.5	0.27	4.35	4.08	2.27	1.15	89.28
NR/tOPA−1	0.31	4.67	4.36	2.13	1.05	92.59
NR/tOPA-3	0.32	4.74	4.42	2.08	1.04	96.15
NR/tOPA-5	0.33	5.00	4.67	1.88	1.02	116.30
NR/tOPA−10	0.36	5.36	5.00	1.72	0.98	135.14

**Table 8:** Rate of vulcanization (k) and activation energies (Ea) of NR/OPA and NR/tOPA with the different loadings.

Rubber Formulation	k	Ea (J/mol)
NR/OPA-0.5	2.133	5781
NR/OPA-1	2.142	5860
NR/OPA-3	2.140	5377
NR/OPA-5	2.154	4940
NR/OPA-10	2.165	4658
NR/tOPA-0.5	2.167	5862
NR/tOPA-1	2.393	5418
NR/tOPA-3	2.406	5300
NR/tOPA-5	2.430	4797
NR/tOPA-10	2.464	4438

**Table 9:** TGA data of NR containing OPA and tOPA at different loading.

Sample	T_onset_ (°C)	T_max_ (°C)	Residue (wt%)
NR	310.3	369.2	99.7
NR/OPA-0.5	312.7	369.6	99.4
NR/OPA−1	317.3	370.4	98.6
NR/OPA-3	321.2	371.3	96.8
NR/OPA-5	325.6	372.1	94.5
NR/OPA−10	331.8	374.5	89.9
NR/tOPA-0.5	313.2	369.7	99.4
NR/tOPA−1	319.2	370.7	98.4
NR/tOPA-3	322.5	371.6	96.7
NR/tOPA-5	327.3	372.8	94.5
NR/tOPA−10	335.5	375.7	89.5

## Data Availability

The data presented in this study are available on request from the corresponding author.
